# Early histopathological changes of secondary degeneration in the spinal cord after total MCA territory stroke

**DOI:** 10.1038/s41598-023-49230-x

**Published:** 2023-12-11

**Authors:** Sarolta Kollai, Dániel Bereczki, Tibor Glasz, Tibor Hortobágyi, Tibor Kovács

**Affiliations:** 1 Department of Neurology, Semmelweis University, Balassa U. 6, Budapest, 1083 Hungary; 2 Károly Schaffer Laboratory of Neuropathology, Department of Neurology, Semmelweis University, Budapest, Hungary; 3 HUN-REN–SU Neuroepidemiological Research Group, Budapest, Hungary; 4 Department of Pathology, Forensic and Insurance Medicine, Semmelweis University, Budapest, Hungary; 5 Institute of Neuropathology, University Hospital Zurich, Zurich, Switzerland; 6 Department of Neurology, Faculty of Medicine, University of Debrecen, Debrecen, Hungary; 7 Department of Old Age Psychiatry, Psychology and Neuroscience, Institute of Psychiatry, King’s College London, London, UK

**Keywords:** Medical research, Neurology

## Abstract

Previous research has not demonstrated secondary degeneration of the spinal cord (SpC) motoneurons after cerebral infarct. The aim of the present study is to investigate the involvement of the anterior horn cells (AHC) in the early post-stroke period using histomorphological and immunohistochemical methods. Post-mortem analysis of the 6th cervical segment was performed in 7 patients who had total MCA stroke within 1 month before death. Nissl-stained sections were used for morphometry, while CD68 and synaptophysin (SYP) immunohistochemistry to monitor microglial activation and synaptic changes in the anterior horn (AH), respectively. Contralateral to the cerebral lesion (contralesional side), cells were smaller after 3 days and larger after 1 week of stroke, especially regarding the large alpha motoneurons. CD68 density increased mainly on the contralesional Rexed’s IX lamina of the SpC. SYP coverage of the large motoneurons was reduced on the contralesional side. Early microglial activation in the AH and electrophysiological signs has suggested the possibility of impairment of anterior horn cells (AHC-s). Our study supported that early microglial activation in the contralesional side of the SpC may primarily affect the area corresponding to the location of large motoneurons, and is accompanied by a transient shrinkage followed by increase in size of the large AHC-s with a reduction of their synaptic coverage. After MCA stroke, early involvement of the SpC motoneurons may be suspected by their morphological and synaptic changes and by the pattern of microglial activation.

## Introduction

Transneural degeneration is a long-established phenomenon in the nervous system, confirmed in many structures by numerous animal and human observations. Most of the human data are from the visual system, where antero- and retrograde degeneration was proved by magnetic resonance (MR) investigations and also by histological studies^1–6^. MR signs of possible secondary degeneration of the ipsilateral mammillothalamic tract and mamillary body in temporal lobe epilepsy and after posterior cerebral artery territory infarction, secondary post-ischemic involvement of the substantia nigra and globus pallidus after striatal infarct, as well as thalamic degeneration after MCA territory infarct had also been reported^7–12^. Hypertrophic olivary degeneration is a form of transsynaptic degeneration caused by a lesion in the Guillain-Mollaret triangle^13^.

The presence of similar changes in the spinal cord (SpC) is still unsettled. Wallerian degeneration of the corticospinal tract following upper motor neuron lesion has been confirmed by pathological and MR studies, traced along the course of the pathway in the internal capsule and brainstem on the same side, and—beyond its decussation in the pyramid of the medulla oblongata—in the contralateral lateral bundle of the SpC^14–17^. Previous research had not found evidence of similar strength for secondary degeneration of the lower motoneurones in the SpC; even experimental animal studies produced conflicting results^18–21^. Prior morphometrical investigations in human cases focused on the chronic phase after suffering a cerebral infarction, while there are no histomorphological studies in the SpC coming from the first month, although electrophysiological signs suggestive of functional impairment of the spinal motoneurons are from this period^22–32^.

Our work focuses on the pathological processes in the cervical SpC in the acute phase (i.e. in the first month) after a total MCA territory stroke, by studying the pattern of microglial activation and the synaptic and morphometric changes in the SpC. Mapping of these early changes may contribute to our understanding of the pathomechanism of residual symptoms and may promote future investigations in the field of neuroprotection and neurorehabilitation. The importance of our study is supported by the high prevalence of stroke worldwide and the large number of patients who live with residual stroke symptoms and disturbing spasticity.

### Patients

Autopsy samples were collected from 7 patients aged between 73 and 85 years who suffered a total unilateral MCA territory infarction proven by computed tomography (CT) imaging on acute admission. Survival times ranged from 3 to 32 days (D3: 3 patients; D6-7: 2 patients; D17: 1 patient; D32: 1 patient) and all patients had severe hemiparesis (Table 1). There was no previous stroke or other central nervous system (CNS) lesion in the medical history or by neuropathological examination and no peripheral nerve injury of the affected upper extremity.

**Table 1:** List of the studied patients and their clinical characteristics.

	Patients	Sex, age	Side of the infarct	Survival (days)
D3	A	M74	R	3
	B	F81	R	3
	C	F85	R	3
D6-7	D	M73	R	6
	E	F79	L	7
D17	F	M80	R	17
D32	G	F85	R	32

## Methods

All methods were carried out in accordance with relevant guidelines and regulations.

**Figure 1:**
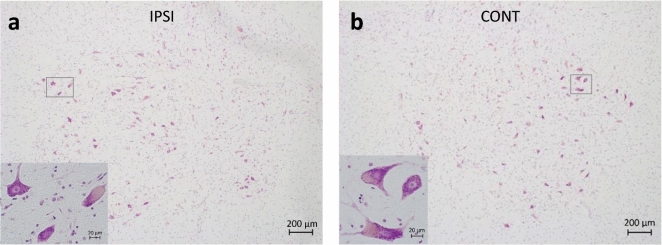
(**a**, **b**) AH-s on the intact (IPSI) (**a**) and on the contralesional (CONT) (**b**) side of the SpC. Large motoneurons are shown by the inserts. Nissl staining.

The autopsies were performed within 48 h after death. The brain and the SpC specimens were removed and fixed in a 10% formal-saline solution for 30 days. Neuropathological examination of the brains was performed (by TH and TK), establishing the total involvement of the MCA territory. The C6 segment of the SpC was dissected and embedded in paraffin wax and 10 um thick transverse sections were made. Every 6th section was stained with the Nissl-staining method (cresyl-violet 0.1%, Reanal Labor, Budapest, Hungary) for morphometrical analysis (Fig. 1), while every 20th and 21st section was immunostained for CD68 (clone KP1, DAKO, Denmark; dilution 1:200) and SYP (clone DAK-SYNAP, Dako, Denmark; dilution of 1:200), respectively, using VECTASTAIN Elite RTU kit (Vector Laboratories, USA) and counterstained with hematoxylin (Reanal Labor, Budapest, Hungary) for nuclear staining to help morphological orientation.

Images were taken using a Zeiss Axio Imager M2 type light microscope with a Zeiss Axiocam 503 color digital camera and image analysis was obtained by using the ZEN 2.6 computer software (Carl Zeiss AG, Germany). At least 12 and 3 sections were used from each case for morphometry and immunohistochemical analysis, respectively.

### Histochemical morphometry

Total number, diameter, perimeter and cross-sectional area of the anterior horn cells (AHC) were measured in the Nissl-stained sections. The AH was defined by the method of Qiu and Terao: an area of the gray matter bounded by a line laid on the ventral sulcus, and another, perpendicular one to it, which runs through the central canal^30,31^. Cells over an area size of 50 µm^2^ with a clearly discernible nucleolus were considered as AHC-s.

### Immunohistochemichal morphometry

CD68 density was observed in the entire area of the AH, in the Rexed's lamina IX and in the area of the lateral corticospinal tract (CST). AH was defined by the same method we used in morphometric analyses, while the area of the Rexed's lamina IX was marked by the connectors between motoneurons with a diameter over 30 µm. The measurements in the CST were carried out in an arbitrarily sized circle (with a radius of 690 µm) defined by the posterior horn and root, the edge of the lateral bundle and a horizontal line laid on the central canal. The measurements were performed in at least 3 different sections in the C6 segment. CD68 density was determined as the size of the immunostained area divided by the size of the total measured area.

SYP density was assessed in the entire AH and in the Rexed's lamina IX (defined as above). We also calculated the SYP-coverage of the individual AHC (the synaptophysin-covered part of the perimeter divided by the total perimeter). Ten randomly selected AHC-s over 700 µm^2^ cross sectional area and with a clearly visible nucleolus were selected on both sides in every subject.

In all measurements described above the two sides of the SpC were compared and the ipsilesional, unaffected side was used as a control. Measurements were performed by SK and TK, with an interrater reliability of 0.92 (measured on AHC morphometry).

### Statistical analysis

Pooled analysis of the cases based on the number of days passed since the stroke event was performed using the Mann–Whitney test. We also divided the neurons into groups of large and small cells, and then compared the number of cells in the two groups with the opposite side by Chi-square test. Based on the size distribution of the cells in the spinal cord, including Rexed’s lamina IX, cells above 700 µm^2^ were considered as large cells, corresponding to the motor neurons in the Rexed’s lamina IX^33^. Cells between 50 and 700 µm^2^ were considered as small ones. The Wilcoxon test was used to compare the total cell numbers of the two sides.

We also compared the CD68 density of the two (ipsilesional and contralesional) AH-s, lateral bundles and Rexed's laminae IX with t-test. CD68 density of the entire AH and that of the Rexed's lamina IX were compared using the Sign and Wilcoxon tests in each case.

The difference of SYP density values between the two AHs and Rexed's lamina IX and SYP coverage ratio of the large AHCs were evaluated with t-test.

Statistical analysis was performed using the TIBCO Statistica software (version 14.0.1.25, Palo Alto, CA, USA).

### Ethics approval

The study was conducted in accordance with the guidelines set by the Declaration of Helsinki and was approved by the Ethical Review committee in Budapest, Hungary (The Regional and Institutional Committee of Scientific and Research Ethics of the Semmelweis University, Budapest, Hungary) (reference number: 79/2007). Due to the retrospective design of the study, patient informed consent was waived by the same Ethical Review committee.

### Consent to participate

Informed consent was not obtained for the present study because patients were selected from cases where an autopsy and histopathological examination was authorized for other medical, family or official reasons and the specimens are anonymized.

## Results

### Morphometry

**Figure 2:**
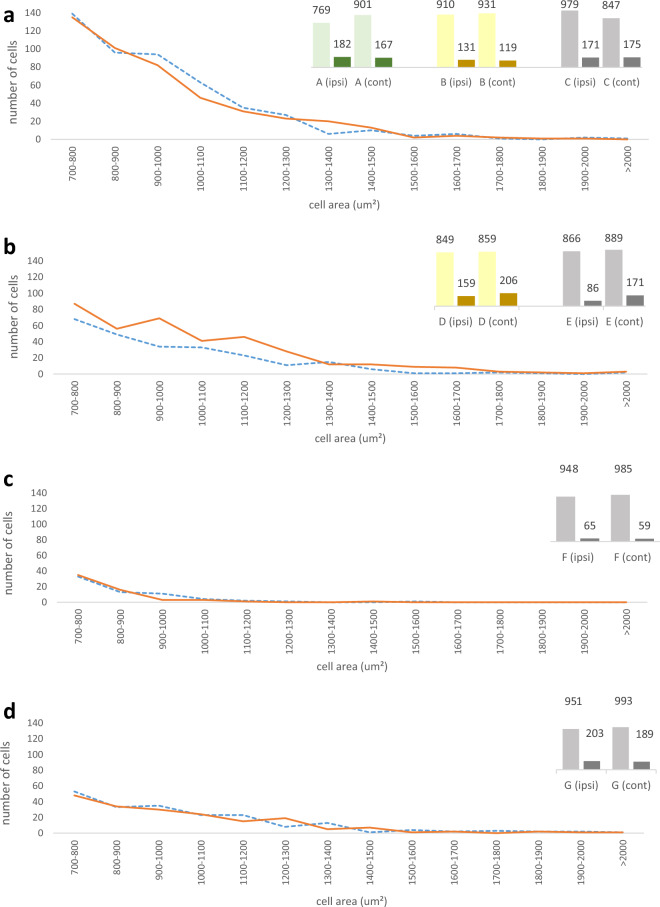
(**a**–**d**) Distribution of cells over 700 um^2^ by cross sectional area (cell area) 3 (**a**), 6–7 (**b**), 17 (**c**) and 32 (**d**) days after total MCA territory stroke. Dotted lines indicate the ipsilesional (IPSI), while continuous ones the contralesional (CONT) side. The right upper part of the images show the number of cells ˂ 700 µm^2^ (light color columns) and 700 um^2^ ˂(dark color columns) on the intact (IPSI) and contralesional (CONT) sides in the individual cases.

Morphometric data did not prove neuronal loss in the contralesional AH of the SpC in the first 30 days after the MCA infarction (*p*: 0.13). In the D3 cases, cells on the contralesional side were significantly smaller, while in the D6-7 cases, they were significantly larger in all three parameters. In D17 and D32 patients, no significant difference was seen between the ipsi- and contralesional sides in these parameters (Table 2, Fig. 2).

**Table 2:** Cell sizes in the AH according the age of the MCA infarct.

	Cell number (n)		Cross sectional area (µm^2^)			Diameter (µm)			Perimeter (µm)		
	IPSI	CONT	IPSI	CONT	*p*	IPSI	CONT	*p*	IPSI	CONT	*p*
D3	3099	3142	403.9 ± 292.8	388.3 ± 290.7	**0.006**	21.3 ± 7.5	20.8 ± 7.6	**0.006**	86.5 ± 37.0	84.9 ± 37.3	**0.006**
D6-7	1961	2125	366.8 ± 287.4	416.9 ± 335.879	** > 0.000**	20.3 ± 7.4	21.5 ± 8.2	** > 0.000**	80.2 ± 34.3	85.4 ± 38.3	** > 0.000**
D17	1013	1044	332.8 ± 207.1	342.9 ± 200.574	0.103	19.6 ± 6.0	20.0 ± 5.9	0.103	80.9 ± 32.2	82.8 ± 30.9	**0.047**
D32	1154	1182	371.5 ± 312.0	387.9 ± 320.630	0.312	20.2 ± 8.0	20.6 ± 8.2	0.312	91.1 ± 60.8	97.1 ± 115.6	0.485

Significant values are in bold.

The proportion of large cells was significantly higher on the contralesional side in the D6-7 cases (D6: *p*:0.0001 and D7: *p*: 0.0330), while no similar shift in the ratio of cell size was revealed in the earlier or later cases (Table 2; Fig. 2).

### Immunohistochemical morphometry

**Figure 3:**
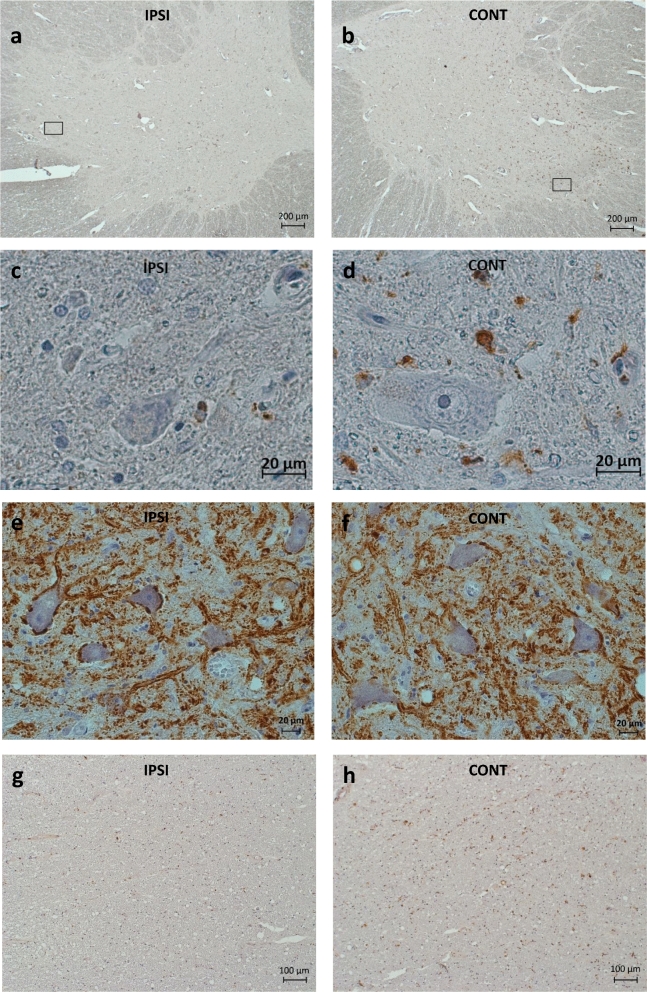
Pathology of the SpC following stroke. (**a**, **b**)Microglial cells (dark dots) in the AH on the intact (IPSI) (**a**) and on the contralesional (CONT) (**b**) side 6 days after MCA occlusion. Black rectangles indicate the parts of the images that are shown at higher magnification in (**c** and** d**). CD68 immunhistochemistry with hematoxylin counterstaining. (**c**, **d**) Microglial cells and processes with high-power magnification in the AH on the intact (IPSI) (**c**) and on the contralesional (CONT) (**d**) side 6 days after MCA occlusion. CD68 immunhistochemistry with hematoxylin counterstaining. (**e**, **f**) SYP-coverage of the AHC-s on the intact (IPSI) (**e**) and on the contralesional (CONT) (**f**) side 3 days after MCA occlusion. SYP immunohistochemistry with hematoxylin counterstaining. (**g**, **h**) Microglial cells (dark dots) in the lateral pyramidal tract on the intact (IPSI) (**c**) and on the contralesional (CONT) (**d**) side 32 days after MCA occlusion. CD68 immunhistochemistry with hematoxylin counterstaining.

CD68 immunohistochemistry (Fig. 3a,b) displayed an outstanding increase in density in the whole contralesional AH (ipsilesional side (mean ± SD): 0.0020 ± 0.0014, contralesional side (mean ± SD): 0.0039 ± 0.0024; n = 21; *p*:0.0008), and this difference was seen already on the third day after MCA occlusion and did not disappear later on. On the contralesional side, CD68 density was higher in the Rexed's lamina IX compared to the whole AH (CD68 density in Rexed’s lamina IX: ipsilesional (mean ± SD): 0.0020 ± 0.0015; contralesional (mean ± SD): 0.0049 ± 0.0031); (Sign test: n:21; *p*:0.0088; Wilcoxon: n:21; *p*: 0.0172), while similar distribution difference could not be observed on the ipsilesional side.

A similar remarkable difference was observed in the CD68 density in the CST in the lateral bundle on the contralesional side compared to the intact ipsilesional side (density ipsilesional (mean ± SD): 0.0024 ± 0.0016; contralesional (mean ± SD): 0.0058 ± 0.0034 *p*: 0.0008) (Fig. 3g, h).

SYP density (all cases) (Fig. 3e,f) was identical on both sides in the complete AH and in the Rexed's lamina IX as well (SYP density ipsilesional side (mean ± SD): 0.2731 ± 0.0878; contralesional side (mean ± SD): 0.2683 ± 0.0870 n:21, *p*: 0.4020), while the SYP coverage (all cases) of the individual motoneurons was significantly decreased on the contralesional side (SYP coverage ipsilesional: mean ± SD: 0.8341 ± 0.1298; contralesional: mean ± SD: 0.7557 ± 0.1499 n:70 *p*:0.0001).

## Discussion

We have found that post-stroke microglial activation on the contralesional side of the SpC primarily affects Rexed’s lamina IX. There was a transient decrease and then an increase in the size of AHCs, mainly affecting neurons above 700 µm^2^ (i.e. large motor neurons). In addition, the synaptic coverage of the surface of large motor neurons diminished during this period.

Based on previous human studies, there is a strong early microglial activation in the lateral bundle and also in the spinal gray matter followed by signs of Wallerian degeneration (WD) in the CST and electrophysiological signs of motoneuron damage as early as on the first day after stroke onset, accompanied by probable cell shrinkage in the AH in the later phase without obvious cell loss^16,17,30,31,34,35^.

Microglial activation follows any injury to the CNS; the consequence of which is partly protective and beneficial by removing tissue debris, but on the other hand it is also detrimental by causing secondary injury to the neurons^36–39^. In cerebral infarction, early microglial activation occurs not only in the area of ischemia and in the descending pyramidal pathway suffering from WD, but also in the AH of the SpC^35^. Our study yielded similar results, a strong presence of CD68 was evident in the whole AH as early as 3 days after stroke and it was significantly higher in the Rexed's lamina IX compared to the entire AH. This difference suggests that the pathology of the spinal motoneurons is more pronounced than the one seen in the interneurons. Since CD68 is a marker of microglial phagocytosis and there is no primary tissue damage in the spinal cord where debris should be removed, it would be reasonable to conclude that microglia may induce secondary synaptic degeneration or reorganization^37,40–42^. To assess this, we observed the density of SYP, a protein component of the presynaptic vesicle membrane, but we did not find a significant decrease in it, although the SYP coverage of the surface of the individual large AHCs is reduced on the contralesional side, suggesting that the synaptic connectivity of the AHCs is affected^43^. These findings should be confirmed by electronmicroscopic studies in the future.

Human electrophysiological studies described distinct electrophysiological signs of lower motor neuron damage in the SpC after upper motoneuron lesion. The earliest electrophysiological signs may be detected from as early as the first day after the stroke and indicate motor unit loss in the paretic muscles, followed by motor unit reorganization^22–25,32,44^. From the second week onwards, spontaneous muscle activity appears, which is no longer present 6 months later^22,26–29^.

These previous electrophysiological studies consistently suggest some kind of transsynaptic dysfunction, but so far, histological analyses have failed to confirm transneural degeneration; no motoneuron loss was demonstrated in the contralesional AH in the C5 segment 6 months after stroke or later, although in this study the mean cross-sectional area of AHC-s was significantly smaller compared to the intact, ipsilesional side^31^. Another study did not find a difference in the number of AHC-s either, although here the L4 segment was examined, whose motoneurons supply the proximal muscles and receive bilateral corticospinal inputs, which in this case may explain the negative result^30^.

Based on our data, the ratio of the large cells increased on the contralesional side 1 week after stroke, preceded by shrinkage of them observed in D3 cases. When the total cell population is examined during the same period, cells appear to be larger on the contralesional side. No similar correlation was found between the later cases, with no difference between the proportion of large and small cells. These may suggest a transient morphological change one week after stroke, with possible cell swelling, affecting mainly cells in the range above 700µm^2^ (i.e. large alpha motoneurons). This might be the morphological consequence of the transsynaptic dysfunction of motoneurons shown by electrophysiological studies^24,32,44^. The increase in size of the neurons could be a compensatory mechanism following decreased or changed synaptic connectivity. Similar mechanisms is proven in Alzheimer’s disease (AD), where the size of hippocampal CA1 neurons is increased in asymptomatic AD cases, compared to cases with mild cognitive impairment or dementia caused by AD^45^.

In D3 cases, cells were significantly smaller in the total AH on the contralesional side, but without change in the ratio of the large and small cells. After one month, no difference was observed between the ipsi- and contralesional side of the SpC, confirming data from previous researches^30,31^.

The interpretation of data from our study is limited by the small number of patients included, but the findings are strengthtened by the strict patient selection and detailed morphometrical analysis. Confirmation in larger studies is necessary, but even these preliminary data suggest that spinal motoneurons undergo morphological changes within the first month after stroke and the identification of the biochemical factors underlying these changes might be helpful in the development of future therapeutic interventions.

## Conclusion

After MCA stroke, early microglial activation in the contralesional side of the SpC primarily affects the Rexed’s lamina IX zone, the area corresponding to the location of large motoneurons. This is followed by transient morphological changes, cell shrinkage and then cell size increase, which is accompanied by a decrease in the synaptic coverage of the motoneurons. .

## Data Availability

All relevant data and materials are available from the authors upon request (TK, corresponding author).

## Abbreviations

AH: Anterior horn
AHC: Anterior horn cell
SpC: Spinal cord
SYP: Synaptophysin
MCA: Middle cerebral artery
CST: Corticospinal tract
CNS: Central nervous system
